# Combination with Stereotactic Body Radiotherapy Offers a Promising Strategy to Overcome Resistance to Immunotherapy in Advanced Renal Cell Cancer

**DOI:** 10.1155/2019/1483406

**Published:** 2019-11-28

**Authors:** Xiaowen Sun, Lu Gan, Aru Na, Lingling Ge, Baoqing Chen, Jiaming Liu

**Affiliations:** ^1^Cancer Center, West China Hospital, Sichuan University, Chengdu, Sichuan Province, China; ^2^Laboratory of Anesthesia and Critical Care Medicine, Translational Neuroscience Centre, West China Hospital, Sichuan University, Chengdu, China; ^3^Department of Obstetrics and Gynecology, West China Second Hospital, Sichuan University, Chengdu, Sichuan Province, China; ^4^West China Hospital, Sichuan University, Chengdu, Sichuan Province, China; ^5^Department of Radiation Oncology, State Key of Oncology in South China, Collaborative Innovation Center for Cancer Medcine, Sun Yat-Sen University Cancer Center, Guangzhou, Guangdong, China; ^6^Department of Urology, Institute of Urology, West China Hospital, Sichuan University, Chengdu 610041, Sichuan Province, China

## Abstract

Immunotherapy for renal cell cancer (RCC) has witnessed several developments for more than two decades. Checkpoint inhibitors, including anti-CTLA-4 and anti-PD-1/PD-L1 blockers, have changed the treatment landscape for patients with advanced RCC in the past 3 years. Despite these advances, more than 55% RCC patients become resistant to different immunotherapies without other treatment combination. Among various attempts at overcoming resistance to immunotherapy, stereotactic body radiotherapy (SBRT) has been found to potentiate the activity of immunotherapy agents through several potential mechanisms, including normalization of microvessels to alleviate tumor hypoxia, improvement in efficient delivery of drugs, abundant neoantigen exposure, and recruitment of antitumor immune cells to alter the immunosuppressive tumor microenvironment. Preclinical studies and clinical case reports have predicted that the combination of SBRT, an immunotherapy, may lead to remarkable results. This review aims to provide the biological basis for the feasibility of combining SBRT to overcome immunotherapy resistance and to review the currently available clinical evidence of this combination therapy in patients with advanced RCC.

## 1. Introduction

Renal cell cancer (RCC) is the third most common urological carcinoma, and over 90% cases of RCC in adults is clear cell in histology [1, 2]. The prognosis of RCC cases depends on the disease stage, tumor properties, the state of tumor metastasis, accurate diagnosis, proper treatment, and so on [2]. Advance and metastatic cases still carry a poor prognosis with a 5-year survival of about 9–12% [3]. Furthermore, nearly 30% of RCC cases with early-stage diagnosis will suffer from recurrence and progression after surgical procedures partly because of pre-existing micrometastatic loci before the surgery or some uncertain reasons [4].

Therapeutic options for advanced RCC patients should be based on histology (clear cell or not clear cell) and the most widely used prognostic factor model is from the Memorial Sloan Kettering Cancer Center (MSKCC) with stratification in three prognostic categories (favorable, intermediate, and poor risk) [5]. Prognostic factors for multivariable analysis included five variables—Karnofsky performance status (KPS) less than 80%, interval from diagnosis to treatment of less than 1 year, serum lactate dehydrogenase (LDH) greater than 1.5 times the upper limit of normal (ULN), corrected serum calcium greater than the ULN, and serum hemoglobin less than the lower limit of normal (LLN). Patients with none of these risk factors are considered low risk or with good prognosis, those with one or two factors present are considered intermediate risk, and patients with three or more of the factors are considered poor risk. First-generation systemic therapy, comprising cytokine-based procedures including interferon-alpha (IFN-*α*) and interleukin-2(IL-2), is recommended for advanced RCC patients since there is documented evidence for its effectiveness against advanced RCC. Targeted therapies including tyrosine kinase (TKI) and mTOR inhibitors, and antibodies against vascular endothelial factor (VEGF) and platelet-derived growth factor (PDGF), have tremendously improved clinical outcomes compared with cytokine therapy alone.

Development and progression of advanced RCC have been slowed or even arrested through immune checkpoint inhibitor (ICI) combination therapy (ipilimumab plus nivolumab), which, in patients with intermediate or poor risk, showed a better overall survival (OS) than VEGF target therapy recently [6]. However, the objective response rate (ORR) is 42% in ICI combination therapy suggesting that most RCC patients are resistant to ICI combination therapy [6]. The lack of predictive biomarkers of high quality has resulted in missed treatment opportunities for RCC patients who could not benefit from ICI therapy. Therefore, it is crucial that RCC patients overcome resistance to treatment and to expand applicable people who could benefit from ICI therapies.

Though RCC was considered to be resistant to radiotherapy, this concept is being challenged, particularly in the past decade, due to the continuous advances and innovation in radiotherapy technology. Increased doses of radiotherapy to tumor lesions has been observed following significant improvement in the accuracy of radiotherapy, which achieved better control of the damage in surrounding normal tissue. Stereotactic body radiotherapy (SBRT), which comprises high doses of radiation delivered in fractions (usually ≤5), has evolved to become an important treatment strategy for both primary lesions and metastatic diseases in different organs for RCC patients. Several key biological pathways triggered by SBRT prime the system immune to eliminate tumor cells. Therefore, SBRT and immunotherapy display synergistic effects, which are reviewed in this study to determine the biological basis and current preclinical and clinical evidence for combination treatment of SBRT and immunotherapy.

## 2. Current Immunotherapy in Clinical Trials for Patients with Advanced RCC

Currently, five immunotherapy agents, IL2, IFN-*α*, ipilimumab, nivolumab, and pembrolizumab, have been approved for treating advanced RCC, either alone or in combination with other drugs. Current immunotherapies for patients with advanced clear cell or non-clear cell RCC are described in Table 1.

IL-2 and IFN-*α* are reported to achieve durable complete or partial response in only a small population of patients [9, 17]. For the majority, the benefit from cytokine-based therapy is limited and the trials to improve the effectiveness have met with efficacy uncertainties. High-dose IL-2 showed substantial toxicity in patients [18]. Thus, selection of patients treated with high-dose IL-2 mainly depends on safety and the tumor histology (clear cell approved), medical comorbidities, patient's performance status, risk scores, and the patient's attitude to treatment risk.

IFN-*α* plus VEGF-targeted therapies such as bevacizumab may improve the prognosis of RCC to a certain degree [10, 11], but whether toxicity was greater in the combination therapy arm remains controversial. However, IFN-*α* alone was inferior compared to the sorafenib (VEGF TKI) [12] or temsirolimus (mTOR inhibitor) monotherapy [13].

Ipilimumab is a selective antibody blocking the interaction between cytotoxic T-lymphocyte antigen 4 (CTLA-4) and its ligands CD80/CD86. Nivolumab selectively blocks the interaction between programmed death-1 (PD-1) and its ligands. The FDA approved nivolumab for previously treated advanced RCC patients. A multicenter phase III trial (CheckMate 214) compared ipilimumab plus nivolumab (ICI combination) followed by nivolumab monotherapy (*N* = 425) versus sunitinib monotherapy (*N* = 422) in patients with advanced RCC [6]. Both groups showed intermediate or poor risk. In comparison with sunitinib, patients receiving ICI had higher ORR (42% vs. 27%, *p* < 0.001), and ICI group showed a significant improvement in complete response (CR) rate (9% vs. 1%, *p* < 0.001) in intermediate- or poor-risk patients. The 18-month OS rate in the ICI group was 75% (95% confidence interval (CI):70–78%), while it was 60% in the sunitinib group [6].

There is controversy over ICI combination therapy in previously untreated favorable-risk patients. Also, the study population in CheckMate 214 included favorable-risk patients treated with ICI combination (*N* = 125) or sunitinib (*N* = 124) [6]. Exploratory analyses of 18-month OS rate found that the favorable-risk patients benefited more from sunitinib (88% vs. 93%). The ORR (29% and 52%; *p* < 0.001) and median progression-free survival (PFS) (14.3 months vs 25.1 months; HR: 2.18; *p* < 0.001) were lower in favorable-risk patients taking ICI combination than sunitinib in this trial. However, the CR rates were 11% and 6% for the ICI combination and sunitinib groups, respectively. Conversely, a phase I trial (CheckMate 016) supported the use of ICI combination in patients at any risk with confirmed advanced clear cell RCC, including those who received prior therapy [14]. The study included patients with poor (*N* = 6), intermediate (*N* = 47), or favorable (*N* = 47) risks. Patients with favorable risk comprised 44.7% of those taking ICI combination. The data for the favorable-risk patients alone were not published, but the 2-year OS for the entire cohort was 67.3%. The confirmed ORR for the cohort was similar in both arms (40.4%) [14]. Because of these conflicting results, the FDA approval for nivolumab plus ipilimumab only included patients with intermediate- or poor-risk RCC for first-line therapy.

In another randomized phase III clinical trial (CheckMate 025), patients (*N* = 821) with previously treated (excluding mTOR inhibitors) advanced clear cell RCC were assigned to receive nivolumab or everolimus (a mTOR inhibitor). The median OS of the nivolumab group and everolimus group were 25.0 months and 19.6 months, respectively. The ORR was also 5 times greater with nivolumab (25% vs. 5%; *p* < 0.001) [15].

Recently, an open-label, randomized phase III clinical trial (KEYNOTE-426) compared the efficacy of pembrolizumab (Keytruda, a PD-1 blocker) plus axitinib (a multitargeted tyrosine kinase inhibitor for VEGFR, c-kit, and PDGFR, *N* = 432) with sunitinib (a multitargeted tyrosine kinase inhibitor for PDGFR, VEGFR, and c-kit, *N* = 429) in previously untreated advanced RCC patients [16]. As a result, 89.9% patients in the pembrolizumab-axitinib group and 78.3% patients in the sunitinib group survived at 12 months in 12.8 months median follow-up. Median PFS durations were 15.1 months and 11.1 months in the pembrolizumab plus axitinib group and in the sunitinib group, respectively (HR 0.69; 95% CI, 0.57–0.84; *p* < 0.001); ORRs were 59.3% and 35.7% in the pembrolizumab-axitinib group (95%CI, 54.5–63.9%) and in the sunitinib group (95%CI, 31.1–40.4%). Regardless of PDL-1 expression, pembrolizumab combined with axitinib benefited patients in all risk groups (favorable, intermediate, and poor risk) [16]. Due to the conspicuous advantage of pembrolizumab plus axitinib over sunitinib on ORR and PFS, the FDA approved pembrolizumab plus axitinib as first-line therapy of all risk groups in advanced RCC on April 19, 2019.

A retrospective analysis of 35 patients with metastatic, non-clear cell RCC who received at least one dose of nivolumab showed that 20% of patients had partial response and 29% of patients had stable disease in 8.5 months median follow-up and 3.5 months median PFS [19]. McKay et al. found that of 43 patients with metastatic, non-clear cell RCC, 8 (19%) patients had modest responses to PD-1/PD-L1 and 4 (13%) patients who received PD-1/PD-L1 monotherapy showed an objective response [20].

In general, the next generation of immunotherapies (ICI: ipilimumab, nivolumab, and pembrolizumab) raised hopes for patients with advanced RCC. From the results reported so far, clear cell RCC and intermediate-risk/poor-risk populations could benefit more than others. ICI therapies showed the potential of improving the ORR and PFS with or without anti-VEGF therapy, which also resulted with lower severe toxicities than high-dose IL2. However, the OS benefit of pembrolizumab plus axitinib over sunitinib remains unknown. Considerable efforts are nevertheless needed to reduce the resistance rate to immunotherapy and improve its efficiency.

## 3. Potential Mechanisms of Adding SBRT to Overcome the Resistance to Immunotherapy

**Figure 1:**
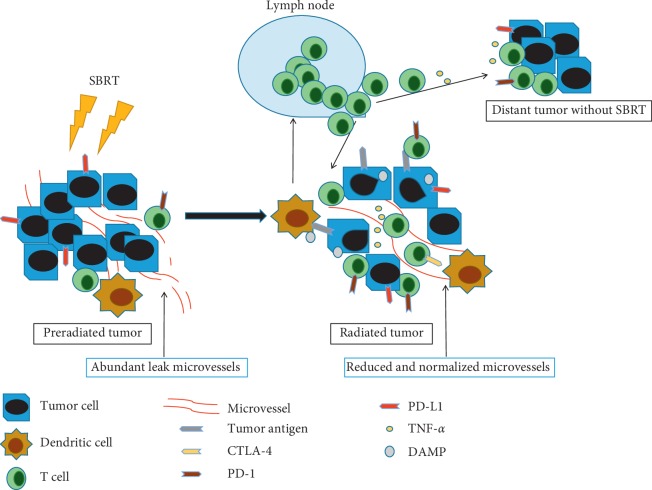
Potential mechanisms of SBRT enhance the efficacy of immunotherapy. SBRT (single dose >8 Gy) reduces and renormalizes the microvessels in tumor. On the other hand, SBRT increases infiltration of antitumor immune cells such as dendritic cells and T cells in the radiated tumor. Theoretically, these antitumor T cells could migrate to the unradiated tumor sites, which is called the abscopal effect. DAMP: damage-associated molecular patterns.

There are several underlying mechanisms explaining how SBRT enhances immunotherapy efficacy in the tumor microvasculature as depicted in Figure 1.

### 3.1. Tumor Microvasculature Response to SBRT

Folkman hypothesized that the most common pathway for new microvessel development in malignant tumor is angiogenesis [21]. In physiological conditions, pro- and antiangiogenic factors maintain a dynamic balance for the normal development of blood vessels. However, in malignant tumors, this balance is perturbed by hypoxia. Excessive proangiogenic factors promote abnormal growth of microvessel, which become disorganized and form tortuous, dilated, hyperpermeable, and dysfunctional microvessels, resulting in intensifying hypoxia and poor transportation efficiency within the tumor microenvironment. These abnormal microvessels impede immune cell migration, function, and transportation of therapeutics. The response of microvessels to SBRT, their normalization structure, and endothelial cell (EC) apoptosis determine the radiosensitivity of certain malignant tumors, including RCC. EC apoptosis might be particularly crucial for RCC because of its extensive microvasculature.

In 2003, Garcia-Barros and colleagues discovered that high-dose SBRT (more than 8–11 Gy) facilitates apoptosis of EC in a dose-dependent manner and normalizes tumor microvasculature [22]. More than 8–11 Gy radiation induced EC apoptosis, and single dose of 15–20 Gy radiation resulted in rapid EC apoptosis. With a single dose of 15 Gy, EC apoptosis, involving acid sphingomyelinase (ASMase), is initiated in one hour, reaches its peak in four hours, and ceases in six hours. ASMase hydrolyses sphingomyelin, a proapoptotic messenger that coordinates transmembrane signaling of FAS-FASL-mediated and tumor necrosis factor- (TNF-) receptor-mediated apoptosis and DR5-TRAIL-mediated apoptosis through death-inducing signaling complexes within seconds after irradiation and without DNA damage. Clustering of receptor-bearing rafts facilitates the stimulation of receptor-mediated apoptosis. Exclusion of survival-regulating proteins and growth factors from these clustered rafts might cause EC apoptosis. A previous study showed that ASMase^−/−^ mice had double the growth rate of MCA129 fibrosarcoma and half the rate of EC apoptosis than ASMase^+/+^ mice [22], suggesting that EC apoptosis plays an important role in tumor cell death. Sathishkumar et al. observed that patients having a complete or partial response after SBRT (15 Gy/1f) had substantially augmented or higher levels of a secretory form of ASMase (S-ASMase) activity before radiotherapy was given (high basal activity), while little-to-no increase in low basal activity was observed in nonresponders [23]. Furthermore, 60% of patients with clear-cell renal cancer are highly vascularized owing to transcriptional silencing (hypermethylation) or mutation of *von Hippel–Lindau (VHL)*. Degradation of hypoxia-inducible factor-1 (HIF-1) requires pVHL, and deficiency in pVHL results in HIF-1 accumulation and angiogenesis.

Given that renal cancer is assumed to be sensitive to SBRT [24], it was found that EC damage appears to be induced by both SBRT and conventional fractionated radiation (CFRT). These contrasting results may be due to the fact that EC apoptosis contributes significantly to tumor cell elimination in SBRT, and EC apoptosis was merely due to low-dose irradiation of CFRT which may not induce tumor cell death effectively, as death signaling in EC is repressed by activation of HIF-1 in tumor cells [25].

Apart from EC apoptosis, SBRT enhances involvement of pericytes in tumor microvessels, and the pericyte-covered microvessels were functional with an increase in perfusion, which could alleviate hypoxia and improve transportation efficacy [26]. Thus, there is a normalization of blood microvessels, offering a “window of opportunity” for immune-cell migration and transportation of therapeutics.

### 3.2. The Systemic Antitumor Effect of SBRT

Basic biological and clinical research in tumor radiotherapy have revealed that local radiotherapy, especially SBRT, can induce systemic antitumor effect in tumor lesions beyond the radiated field, termed the abscopal effect, which has been reported in various malignancies including melanoma, lymphoma, neuroblastoma, and RCC and particularly in pulmonary metastases. A valid hypothesis explaining the mechanism behind abscopal effect is that high-dose radiation can cause tumor cells to die within a short period and expose new tumor antigens, so that radiated tumor cells function as natural tumor vaccines after radiation exposure [27–29]. Concurrently, during the process of tumor cell death, damage-associated molecular patterns, such as HMGB1, ATP, and heat shock proteins, are also released in large quantities. These molecules can effectively induce dendritic cells (DCs) to recognize tumor-specific antigens resulting in their capture and migration of DCs to draining lymph nodes, where tumor antigens are presented to T cells [30], which in turn get activated and undergo massive proliferation. Activated effector T cells enter the circulatory system, recognize tumor cells far from the radiated field, and exert antitumor effects [31, 32].

To explore whether SBRT can enhance the expression of tumor-associated antigens in patients with advanced RCC, Singh et al. studied the response to SBRT in patients with advanced RCC. This study evaluated patients receiving neoadjuvant SBRT following surgery and found SBRT patients had higher expression of tumor-associated antigens (MUC-1, CA-9, 5T4, and NY-ESO-1) and costimulatory molecules ICAM-1 and CD80 compared with patients without SBRT [33]. Moreover, the apoptosis inducers TNF-*α* (24–72 h after SBRT), IL-1*α*, IL-1*β*, IL-6 FASL, and TGF-*β* were released during radiotherapy; higher levels of TNF-*α* agreed with the abscopal effect and complete tumor response [23, 34].

## 4. Efficacy of SBRT in Patients with Advanced RCC

Results from several studies support that SBRT differs from CFRT for RCC patients, and SBRT is effective at controlling both primary and metastatic lesions of RCC, as summarized in Table 2.

### 4.1. SBRT Differs from CFRT in Treating Patients with RCC

In recent years, SBRT has been delivered to patients with advanced RCC, with results showing a slow but persistent shrinkage of the renal tumor after SBRT [50]. Compared with CFRT, in RCC with bone metastasis, the median time to symptom relief between SBRT and CFRT was similar, but the symptom control rates of SBRT were much higher than those of CFRT [35]. Furthermore, the authors of the study also showed that the biologically effective dose (BED) ≥80 Gy was significant for better clinical response and was predictive of local control [35]. Similar results were reported by Altoos et al. showing SBRT-mediated control of thoracic, abdominal, and soft tissue lesions in RCC, with predictive factors for better local control being BED ≥100 Gy and dose per fraction ≥9 Gy [36]. An analysis of radiographic and symptomatic RT responses in 27 consecutive RCC patients with 37 lung lesions found that rates of radiographic local control with SBRT were much higher than CFRT [37]. To explore the difference between SBRT and CFRT on spine metastases from RCC, a total of 110 patients (34 CFRT; 76 SBRT) were retrospectively analyzed [51]. The researchers found that both CFRT (20 Gy/5f) and SBRT (15 Gy/1f) provided effective relief of symptomatic spine metastases from RCC, whereas CFRT relieved pain faster, and pain relief with SBRT was more durable [51].

### 4.2. SBRT Is Effective in Controlling Primary Renal Lesions

Results from several studies indicate that SBRT is effective in controlling primary renal lesions. For example, renal tumors treated with SBRT show significant reductions in growth rate and tumor size after radiation [52]. Furthermore, a prospective phase I trial suggested that SBRT might be an alternative to cytoreductive nephrectomy for inoperable patients with advanced RCC [39]. The median tumor size was increased 17.3% at 5.3 months, and the median OS was increased at 6.7 months [39]. Inadequate single doses (≤7 Gy) in this prospective study could be the reason for these moderate results. For asynchronous bilateral RCC (*N* = 9), SBRT resulted in an ORR of 55.6%, and the 1-, 3-, and 5-year OS rates were 66.7%, 53.3%, and 35.6%, respectively [40]. Among patients with localized RCC who were not suitable for surgery, a phase I study using SBRT (24–48 Gy/4f) showed three partial responses and 12 patients with stable disease among those with an evaluable response (*N* = 15) [41]. Siva et al. applied SBRT (26 Gy/1f for tumors <5 cm or 42 Gy/3f for tumors ≥5 cm) on inoperable primary kidney cancers and found freedom from local (100%) and distant (89%) progression, with an overall 2-year survival rate of 92% [42]. However, SBRT led to dose-dependent renal dysfunction at 1- and 2-years [42]. Therefore, sparing functional kidney from high-dose irradiation regions might help reduce the risk of renal dysfunction. In this context, SBRT (48 Gy/3f) was found to be effective for primary small renal tumors and results in a satisfactory local control rate [43].

### 4.3. SBRT Controls Intracranial and Extracranial Metastases in RCC

At present, several early studies have demonstrated that SBRT has an inhibitory effect on RCC metastases, including intracranial and extracranial metastases.

#### 4.3.1. Intracranial Metastases Controlled by Stereotactic Radiosurgery (SRS)

Brain metastasis (BM) usually indicates poor prognosis in patients with RCC. Whole brain radiation therapy (WBRT) is considered a standard treatment in patients with multiple (>5) BMs. However, WBRT (usually 2–3 Gy per fraction) has limited efficacy in patients with BM from radio-resistant tumors such as RCC and melanoma whose median survival is 2–4 months. Stereotactic radiosurgery (SRS) for BM from RCC has been regarded as an alternative to surgery and delivers high-dose radiation in no more than 3 fractions (usually only one fraction), but avoids the toxic effects of WBRT. Studies in this regard have shown local control in 24 of 32 renal patients with 52 metastases while 4 patients had local progression using SRS for brain metastases in patients, in which the median dose was 22.0 Gy (range, 12.8–24.0 Gy), and the median OS was 6.3 months (range, 0.4–100.4 months) [44]. To evaluate outcomes of SRS in 16 RCC patients with multiple (≥5) simultaneous BMs (99 lesions in total) treated with SRS showed OS after 6 months and 1 year to be 50% and 31%, respectively. The median OS was 7.1 months (range 1–21), and 91% patients were free from local failure [45]. Besides, it has been found that SRS dose >18 Gy was associated with improved survival in patients with RCC [53]. Using this dose (range 15–20 Gy), a study involving 81 patients treated with SRS for BM from melanoma or RCC showed actuarial OS rates at 6 months and 1 year of 55.4% and 30.2%, respectively, and one-year local control (LC) rate of 79.4% for RCC [46]. Another similar, but smaller, study involved BM from melanoma (*N* = 26) or RCC (*N* = 15) patients, which found the lack of statistical significant differences in OS between patients with RCC and melanoma (8.4 mo vs 5.0 mo, *p*=0.11) [54].

The results of these studies indicated that the OS of patients with BM from RCC treated with SRS is about 6.3–8.4 months, which is much longer than patients who underwent WBRT. The lack of high-grade evidence in current retrospective studies warrants the need for prospective studies in order to guide clinical practice, with the inclusion of more numbers of BMs to make valid conclusions.

#### 4.3.2. Extracranial Metastases Controlled by Stereotactic Radiosurgery (SRS)

The ability of SBRT to control extracranial metastases in RCC was demonstrated in recent studies on 84 patients with 175 metastatic extracranial lesions who received SBRT (40–60 Gy/5f or 30–54 Gy/3f or 20–40 Gy/1f); the 1-year local control (LC) rate after SBRT was 91.2%, and one factor of local failure was BED <115 Gy [48]. Another retrospective study of 48 patients treated for 70 spine metastases showed that the spine recurrence rates of 60% were mainly associated with salvage SBRT, which was only 20% for upfront SBRT. The study suggested that an early SBRT with higher doses could be more effective than salvage SBRT [49]. As mentioned above, SBRT effectively relieves symptomatic spine metastases in RCC. Compared with CFRT, SBRT trends to produce more durable pain relief [51], as demonstrated in 57 RCC patients (88 treatment) with spine metastasis, wherein Balagamwala et al. found that a single fraction SBRT achieved a median survival of 8.3 months and relieved pain rapidly with a median duration of 5.4 months of pain relief [38].

The currently available evidence reviewed in this study suggests that SBRT alone is effective for RCC, including primary lesions treatment and intracranial and extracranial metastases control; especially, patients with multiple intracranial metastases face poor prognosis. Single dose <7 Gy might be ineffective to achieve satisfactory treatment results in RCC patients, but higher dose radiation in SBRT monotherapy exerted robust disease control with acceptable clinical risk.

## 5. Preclinical and Clinical Evidence for the Inclusion of SBRT to Overcome Resistance to Immunotherapy

### 5.1. Preclinical Evidence

The introduction of ICIs, initially with anti-CTLA-4 antibodies, initiated a revolution in oncology. The inclusion of radiotherapy to ICI, animal models, or clinical studies focusing on the integrating radiation and related drugs followed in an attempt to find effect of radiotherapy on immune activation in several solid tumors [55]. Under this strategy, combining radiation with immune-checkpoint blockade increased locoregional control of tumors [31, 56]. Furthermore, combination of local radiation with anti-CTLA-4 and anti-PD-1/PD-L1 inhibitors increased systemic disease control mediated by the abscopal effect [57]. An increase in complete regression of the irradiated primary tumor and reduced size of nonirradiated tumors outside the radiation field were observed when SBRT was combined with PD-1 blockade in melanoma and RCC models [58]. This effect was not attributed to tumor histology or host genetic background, but as it was tumor-specific, the effect was potentiated by PD-1 blockade, an abscopal tumor-specific immune response induced by radiotherapy in nonirradiated tumors [58]. The abscopal effect was exerted only in a small proportion of patients who received anti-CTLA-4 combined with radiotherapy, leading to PD-1/PDL1-mediated resistance to ipilimumab [57]. Another study showed blockade of adaptive immune resistance mediated by anti-PD-1/PDL1 antibodies upon localized radiation with anti-CTLA-4 therapy. Furthermore, nonredundant immune mechanisms mediated the superior activity of radiation and dual immune checkpoint blockade [59].

### 5.2. Clinical Evidence

Clinical evidence reporting combination of SBRT with immunotherapy in advanced RCC is scant. A phase-2 trial combining high-dose IL2 and SBRT in patients with metastatic RCC [60] showed that 1–3 lesion sites were treated with SBRT with a dose of 21–27 Gy for single fraction or 25–33 Gy for 3 fractions. The primary endpoint of the study—response rate—was 40%, with 1 patient presenting CR and 3 patients showing PR. The median duration of overall response (including CR and PR) was 5 months, and median stable disease (SD) duration was 6 months. Addition of SBRT to IL-2 increased the response rate in metastatic RCC patients by about 2-folds compared with IL-2 alone. Two cases have reported the induction of abscopal effect when SBRT was combined with ICI therapy in advanced RCC patients. One case reported by Xie et al. showed a systemic complete response to SBRT (32 Gy/4f) and pembrolizumab (anti-PD-1 antibody) in a patient with metastatic RCC [61]. The metastatic lymph nodes in the left mediastinum were irradiated with a total of 32 Gy administered in four fractions on four consecutive days [61]. The second case was that of a 24-year-old male with advanced clear-cell RCC and bone, lung, and nodal metastases who received SBRT (27 Gy/3f) to the sacrum metastatic mass and subsequent ipilimumab and nivolumab therapy [62]. The sacrum mass was obviously shrunk with the therapy and no radiological evidence for lung and nodal metastases was found more than 12 months after SBRT [62].

To determine the effect of combining SBRT with immunotherapy, we searched ClinicalTrials.gov for studies and identified 13 ongoing clinical trials (Table 3). The vast majority of these trials were phase-2 studies and combined ICIs and (or) high-dose IL2.

## 6. Discussion

Recently, a single-arm phase-2 trial, which combined SBRT and a PD-1 blocker (pembrolizumab), suggested PFS improvement without serious safety signals in patients with oligometastatic NSCLC [63]. Immunotherapy (especially ICI) offers hope for patients with advanced RCC, particularly when SBRT is offered in combination. High dose of radiation effectively results in abundant ECs apoptosis which aids in reducing and renormalizing microvessels in the tumor for better transportation of therapeutics and migration of immune cells. Furthermore, SBRT has the potential to prime the immune system by exposing a mass of tumor antigens after irradiation. We acknowledge that there is limited evidence regarding this hypothesis and additional clinical studies are needed. However, in our humble opinion, SBRT offers a promising strategy for overcoming the resistance to immunotherapy in advanced RCC. Nevertheless, limitation of the combined therapy exists as follows:

First, there exists the possibility of severe treatment-related adverse events. High-dose IL2 itself has shown to induce substantial toxicity. Furthermore, ICI therapy-induced acute kidney injuries such as acute tubulointerstitial nephritis, acute interstitial nephritis, and increased blood creatinine or acute renal failure have been reported [64–66]. As mentioned previously, application of SBRT to renal primary lesions could lead to dose-dependent renal dysfunction. Therefore, the combination of SBRT with PD-1/PD-L1 inhibitors will probably increase therapy-associated severe adverse events. Moreover, the incidence of other common treatment-related adverse events such as hypothyroidism and hyperthyroidism, which were the most frequent endocrine immune-related adverse events for PD-1/PD-L1 inhibitor alone, must be considered [67].

Second, the dose, fractions, and targets of SBRT plan are crucial, whereas a single dose <8 Gy might be insufficient and a higher dose presents a higher risk, particularly when combined with immunotherapy. Therefore, a dose-escalation study is warranted to maximize clinical efficacy with acceptable toxicities for prospective clinical trials. Encouraging results from preclinical and clinical studies support the synergistic effect of SBRT and ICI therapy against brain metastases from melanoma [68, 69]. Marrow-derived suppressor cells (MDSC) and immunosuppressive B cells could impede the antitumor activity induced by SBRT and immune therapy [70, 71]. These immunosuppressive cells and heterogeneity in tumor might be the reasons for low incidence of abscopal effect in the clinic. Brooks and Chang suggested that we should abandon single-site radiation and that radiotherapy could be delivered to all targetable disease sites to broaden the T-cell repertoire and maximize the activation of the immune response [72].

Third, an appropriate sequence of SBRT and immunotherapy should be planned with detailed consideration. Harris et al. reported that the highest antitumor immune response in the mouse model of prostate cancer was obtained by adding immunotherapy after 3–5 weeks of radiotherapy; however, there was no obvious antitumor immune response after the end of radiotherapy [73]. It has also been suggested that CTLA-4 antibodies should be used to deplete regulatory T cells prior to radiotherapy to obtain maximum immune effects [74].

Fourth, pembrolizumab plus axitinib have yielded outstanding results, suggesting the benefit of concurrent or sequential treatment with anti-VEGF therapy combined with SBRT and immunotherapy, especially for patients with multiple lesions, some of which may be unsuitable for SBRT. However, the potential toxicities of anti-VEGF therapy with SBRT and immunotherapy need more attention [75].

In conclusion, combination of SBRT with immunotherapy may unlock antitumor immune responses that have the potential of overcoming resistance to immunotherapy in patients with advanced RCC.

## Figures and Tables

**Table 1:** Main clinical trials of immunotherapy for advanced RCC.

Type of RCC	Drug	Phase	No. of pts	Line of therapy	ORR	mPFS (month)	mOS (month)	Reference
Undifferentiated	High-dose IL2	2	71	ND	ORR = 17% CR = 5.6%	NA	15.5	Atkins et al., [7]
Undifferentiated	High-dose IL2	3	96	ND	ORR = 23.3% CR = 8.4%	14	17.1	McDermott et al., [8]
Undifferentiated	IL2 plus IFN*α*-2a	3	140	ND	ORR = 13.6% CR = 3.5%	NA	17	Negrier et al. [9]
Clear cell	Arm 1: bevacizumab plus IFN*α*-2a; Arm 2: IFN*α*-2a	3	325 289	ND	Arm 1: ORR = 31% CR = 1%; Arm 2: ORR = 13% CR = 2%	10.2 5.4	18.3 17.4	Escudier et al. [10]; Rini et al. [11]
Clear cell	IFN*α*-2a	2	189	First line	ORR = 39% CR = 2%	5.6	NA	Escudier et al. [12]
Both clear cell and non-clear cell enrolled	Arm 1: temsirolimus; Arm 2: IFN*α*-2a; Arm 3: both	3	209 207 210	First line	Arm 1: ORR = 8.6%; Arm 2: ORR = 4.8%; Arm 3: ORR = 8.1%	Arm 1: 3.8; Arm 2: 1.9; Arm 3: 3.7.	10.9 7.3 8.4	Hudes et al. [13]
Clear cell	Nivolumab (N) plus ipilimumab (I)	1	N3I1 = 47; N1I3 = 47	First line	Both ORR = 40.4% in the N3I1 and N1I3 arms; CR = 10.6% in the N3I1 arm and none in the N1I3 arm.	N3I1 = 7.7; N1I3 = 9.4	Not reached in the N3I1 arm and 32.6 months in the N1I3 arm	Hammers et al. [14]
Clear cell	Arm 1: nivolumab plus ipilimumab Arm 2: sunitinib	3	550 546	First line	Arm 1: ORR = 42%; CR = 9%; Arm 2: ORR = 27%; CR = 1%;	Arm 1: 11.6; Arm 2: 8.4.	Not reached in arm 1 and 26 months in arm 2	Motzer et al. [6]
Clear cell	Arm 1: nivolumab Arm 2: everolimus	3	821	Second line or third line	Arm 1: ORR = 25%; CR = 1%; Arm 2: ORR = 5%; CR < 1%;	Arm 1: 4.6; Arm 2: 4.4.	Arm 1: 25; Arm 2: 19.6	Motzer et al. [15]
Clear cell	Arm 1: pembrolizumab plus axitinib Arm 2: sunitinib	3	432 429	First line	Arm 1: ORR = 59.3%, CR = 5.8%; Arm 2: ORR = 35.7%, CR = 1.9%;	Arm 1: 15.1; Arm 2: 11.1.	Not reached in both arms	Rini et al. [16]

RCC: renal cell cancer; pts: patients; ND: not demanded; ORR: objective response rate; mPFS: median progression-free survival; mOS: median overall survival; IL2: interleukin-2; CR: complete response.

**Table 2:** SBRT is effective in primary lesions and metastases of advance RCC.

Study type	No. of patients/lesions	SBRT target	SBRT regimen	Local control	OS	AE (≥Grade 3)	Ref
Retrospective study	50 lesions	Bone metastasis	Most common is 27 Gy/3f	Rates at 12 and 24 months were both 74.9%	NA	Grade 3 AE: 1 patient, dermatitis	Amini et al. [35]
Retrospective study	36 lesions	Thoracic, abdominal, and soft-tissue lesions	Most common is 50 Gy/5f	Rates at 12, 24, and 36 months were 100%, 93.41%, and 93.41%, respectively	Median OS about 32 months	Grade 3 AE: 1 patient, mucositis	Altoos et al. [36]
Retrospective study	27 pts/37 lesions	Lung metastasis	Median SBRT dose and fraction were 50 Gy (range 25–60) and 3 (range 1–6)	92.3% for median follow-up 16 months	NA	0	Altoos et al. [37]
Retrospective study	57 pts/88 lesions	Spinal metastases	Single fraction, median 15 Gy	Median 26 months	8.3 months (1.5–38)	0	Balagamwala et al. [38]
Prospective phase I trial	12 pts	Primary renal lesions	25 Gy, 30 Gy, or 35 Gy in 5 fractions	NA	6.7 months (1.5–16.4)	Grade 3 AE: 3 patients, fatigue (2) and bone pain (1)	Correa et al. [39]
Retrospective study	9 pts	Bilateral primary renal lesions	60–85 Gy was delivered at 5–7 Gy/fraction	Rates at 1, 3, and 5 years were 64.8, 43.2, and 43.2%, respectively	Rates at 1, 3, and 5 years were 66.7, 53.3, and 35.6%, respectively	0	Wang et al. [40]
Prospective phase I trial	15 pts	Primary renal lesions	24–48 Gy/4f	100% for median follow-up 13.67 months	Estimated 3-year OS post-treatment was 72%, 95% CI (0.44–0.87)	Grade 4 AE: 1 patient (5.3%) with duodenal ulcer possibly treatment-related	Ponsky et al. [41]
Prospective study	37 pts	Primary renal lesions	26 Gy/1f for tumors <5 cm and 42 Gy/3f for tumors ≥5 cm	Rates at 2 years was 100%	Rates at 2 years were 92%	Grade 3 AE: 1 patient (3%).	Siva et al. [42]
Retrospective study	21 pts	Primary renal lesions	48 Gy/3f	Rates at 1 year and 2 years were 92 and 84%, respectively	Rates at 1 year and 2 years were both 95%	0	Kaplan et al. [43]
Retrospective study	32 pts/52 lesions	Brain metastasis	22.0 Gy (range, 12.8–24.0 Gy)	NA	6.3 months (0.4–100.4 months)	NA	Shah et al. [44]
Retrospective study	16 pts/99 lesions	Brain metastasis (≥5)	SRS	91% of targets	50% after 6 months and 31% after 1 year	NA	Mohammadi et al. [45]
Retrospective study	81 pts/117 lesions	Brain metastasis (from melanoma or renal cancer)	18 Gy (range 15–20 Gy)	Rate at 1 year was 79.4% for renal cancer	Rates at 6 months and 1 year were 55.4% and 30.2%, respectively	NA	Feng and Lemons et al.
Retrospective study	15 pts	Brain metastasis	SRS	NA	8.4 months	NA	Feng et al. [46]
Retrospective study	18 pts/39 lesions	Oligometastatic renal cancer (extracranial)	8–14 Gy *∗* 3 fractions or 4–5 Gy *∗* 10 fractions	Rate at 2 years was 91.4%	2 years was 85%	NA	Ranck et al. [47]
Retrospective study	84 pts/175 lesions	Extracranial metastasis	(40–60 Gy/5f or 30–54 Gy/3f or 20-40 Gy/1f	1-year LC rate was 91.2%	NA	Grade 3 events: 8 patients (4.6%).	Wang et al. [48]
Retrospective study	48 patients/70 lesions	Spinal metastases	NA	Rate at 21 months was 72%	66 months (CI95% 54–79)	NA	Serrand et al. [49]

NA: not available; pts: patients; OS: overall survival; AE: adverse effect; SRS: stereotactic radiosurgery.

**Table 3:** Ongoing clinical trials which combined SBRT and immunotherapy in advanced RCC.

Identifier	Phase/no. of patients	Status	Cancer	Immunological agents	Schedule of SBRT	Line of therapy
NCT03469713	NA/68	Recruiting	Metastatic RCC	Nivolumab	30 Gy in 3 consecutive fractions	II-III
NCT01884961	II/35	Recruiting	Metastatic melanoma or RCC	High-dose IL2	Three daily doses of SBRT at 6–12 Gy to at least 1 and up to a maximum of 5	NA
NCT02855203	NA/30	Recruiting	Oligometastatic renal tumors	Pembrolizumab	18–20 Gy in 1 fraction	≤III
NCT01896271	II/26	Active, not recruiting	Metastatic RCC	High-dose IL2	8Gy–20 Gy in 1–3 fractions	NA
NCT02306954	II/84	Recruiting	Metastatic RCC	High-dose IL2	40 in 2 fractions	NA
NCT03065179	II/25	Recruiting	Metastatic RCC with a clear-cell component	Nivolumab and ipilimumab	NA	Not limited
NCT02781506	II/35	Recruiting	Metastatic RCC	Nivolumab	Dose variable in 1–3 fractions	≥II
NCT03050060	II/120	Recruiting	Metastatic/recurrent RCC/recurrent melanoma/recurrent NSCLC	Nelfinavir mesylate, pembrolizumab, nivolumab, and atezolizumab	Image-guided hypofractionated radiotherapy	Not limited
NCT03115801	II/112	Recruiting	Metastatic RCC	Nivolumab	30 Gy in 3 fractions	Not limited
NCT03474497	I-II/45	Not yet recruiting	Metastatic NSCLC/metastatic melanoma/RCC/head and neck squamous cell carcinoma	IL-2, pembrolizumab	24 Gy in 3 fractions delivered on consecutive or every other day	NA
NCT03693014	II/60	Recruiting	Metastatic cancer: melanoma/lung cancer/bladder cancer/RCC/head and neck cancers	Nivolumab for RCC	Image-guided, 27 Gy in 3 fractions	NA
NCT03511391	II/97	Recruiting	Urothelial carcinoma/melanoma/RCC/NSCLC/head and neck cancer	Pembrolizumab or nivolumab	24 Gy in 3 fractions	II for RCC
NCT02978404	II/60	Recruiting	Brain metastases of metastatic clear-cell RCC or metastatic NSCLC	Nivolumab	15–20 Gy in 1 fraction	≤IV

NA: not available; NSCLC: non-small-cell lung carcinoma; RCC: renal cell cancer.
